# A Rare Case of Perforated Caecal Cancer Disguised as a Strangulated Right Inguinoscrotal Hernia

**DOI:** 10.7759/cureus.59862

**Published:** 2024-05-08

**Authors:** Andrianos-Serafeim Tzortzis, Kyriaki Grylli, Nikolaos Koliakos, Ilias Kagkouras, Agamemnon Kokkofitis, Leonidas Lampropoulos

**Affiliations:** 1 ENT, Lister Hospital, East and North Hertfordshire NHS Trust, Stevenage, GBR; 2 General Surgery, General Panarcadian Hospital of Tripolis, Tripolis, GRC; 3 Abdominal Surgery, Erasme Hospital, Free University of Brussels (ULB), Brussels, BEL

**Keywords:** inguinoscrotal hernia, hernia repair, emergency surgery, hernia surgery, bowel cancer

## Abstract

Hernia repair surgery is among the most common procedures performed worldwide. Bowel cancer is the third most common cancer. However, bowel cancer coexisting within an inguinal hernia is extremely rare. In this report, we discuss a rare case of a 72-year-old male patient who presented with perforated caecal cancer within a strangulated right inguinoscrotal hernia.

## Introduction

Inguinal hernia repair is one of the most commonly performed procedures worldwide, with more than 20 million repairs reported globally each year [1]. Hernia repair can be performed with an open approach, endoscopically, or robotically [2]. These repairs are generally performed on an elective basis, to prevent complications such as incarceration and strangulation [3].

Large bowel cancer is the third most common cancer globally and the cancer type with the second highest mortality; it accounts for 1/10 cases of cancer worldwide [4,5]. The mainstay of treatment in early-diagnosed cases is surgery; however, in 25% of the cases, the disease has already progressed and metastasized, rendering surgery noncurative [4]. The coexistence of colon cancer within the sac of an inguinal or inguinoscrotal hernia is considered an extremely rare entity [6]. We present a case of an adult male with a strangulated right inguinoscrotal hernia, which contained a perforated cecal adenocarcinoma; he was treated with right hemicolectomy and herniorraphy.

## Case presentation

A 72-year-old male presented to the emergency department complaining of generalized weakness for five days. He had experienced unintentional weight loss over the last three months but did not report any changes in his bowel habits or any presence of blood in his stools. He was not on any regular medication and had never undergone any surgeries or upper or lower GI endoscopy.

His vitals were mostly normal, except for a raised temperature of 37.5 °C and a heart rate of 95 bpm. The rest of the clinical examination was unremarkable. The chest X-ray was normal. However, during the abdominal examination, a large right inguinoscrotal hernia was found (20 x 25 cm). The hernia was firm on palpation, irreducible, and slightly painful, and the skin of the scrotum was erythematous and warm. When asked, the patient mentioned that the hernia had been present for a decade, but became irreducible a year ago. The blood tests revealed increased neutrophil counts (18.5 x 10^9^/L), raised inflammatory markers (CRP: 17mg/dL ), and anemia (Hb: 117 g/L), as shown in Table 1.

**Table 1:** Hematological parameters of the patient CRP: C-reactive protein; Hb: hemoglobin

Parameter	Patient value	Reference range	Units
White cell count	18.5	3.6-11.0	x 10^9^/L
Hb	117	130-180	g/L
CRP	17	0.0-0.7	mg/dL

**Figure 1:**
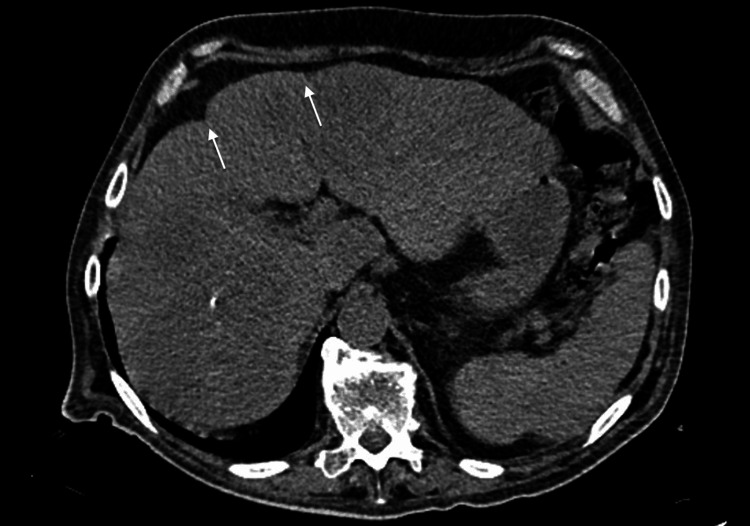
CT scan showing the liver with irregular contour (white arrows) CT: computed tomography

**Figure 2:**
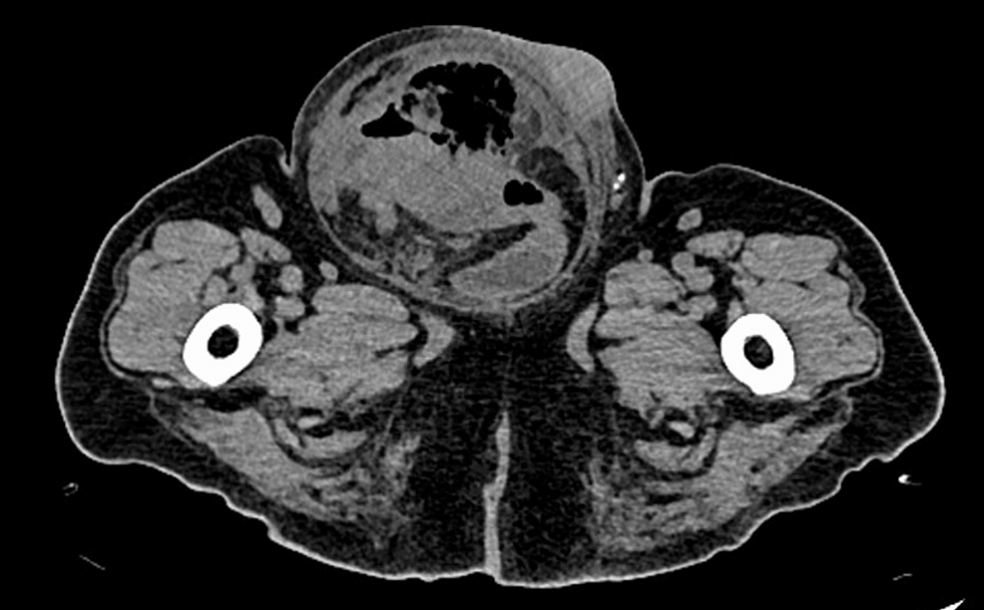
CT scan showing a right inguinoscrotal hernia with a mass within the bowel lumen and free air inside the hernia CT: computed tomography

An abdominal CT was performed, which showed the liver with an irregular contour (Figure 1), a mass within the bowel lumen, and free air inside the hernia (Figure 2). A hernia complicated by bowel rupture was suspected and the patient was immediately taken to the operating room.

During the operation, a right-sided inguinal incision was performed. The hernia sac was identified and opened, and the caecum, part of the ascending colon, and the terminal ileum were included in the scrotal hernia. There was a free wall rupture within the hernia sac containing exudative fluids and fecal content. Macroscopically, the rupture was located in the caecum, where a firm and irregularly shaped mass was palpated inside its lumen. A midline laparotomy incision was then made and the patient underwent right hemicolectomy and ileotransverse anastomosis. In addition, the right testis, the epididymis, and the spermatic cord were resected due to the significant contamination of the scrotum, the proximity of the testis to the tumor, and the macroscopic impression that the testis had been invaded. During the investigation of the rest of the abdomen, multiple firm nodules were palpated on the surface of the liver. Finally, a tension-free Shouldice repair was performed. No mesh was used.

**Figure 3:**
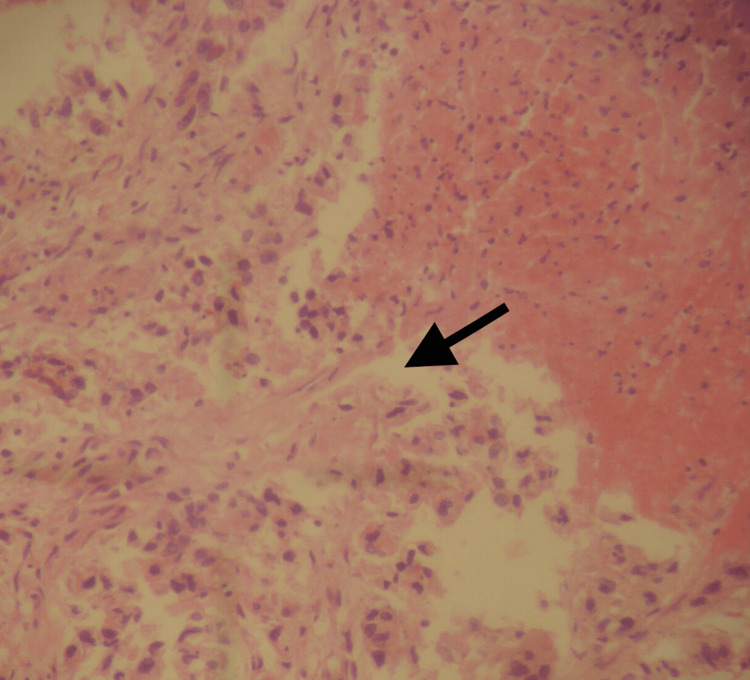
Histological image of the ruptured area of the intestinal wall The shoots of carcinoma are seen on the left (arrow) (H & E x20)

**Figure 4:**
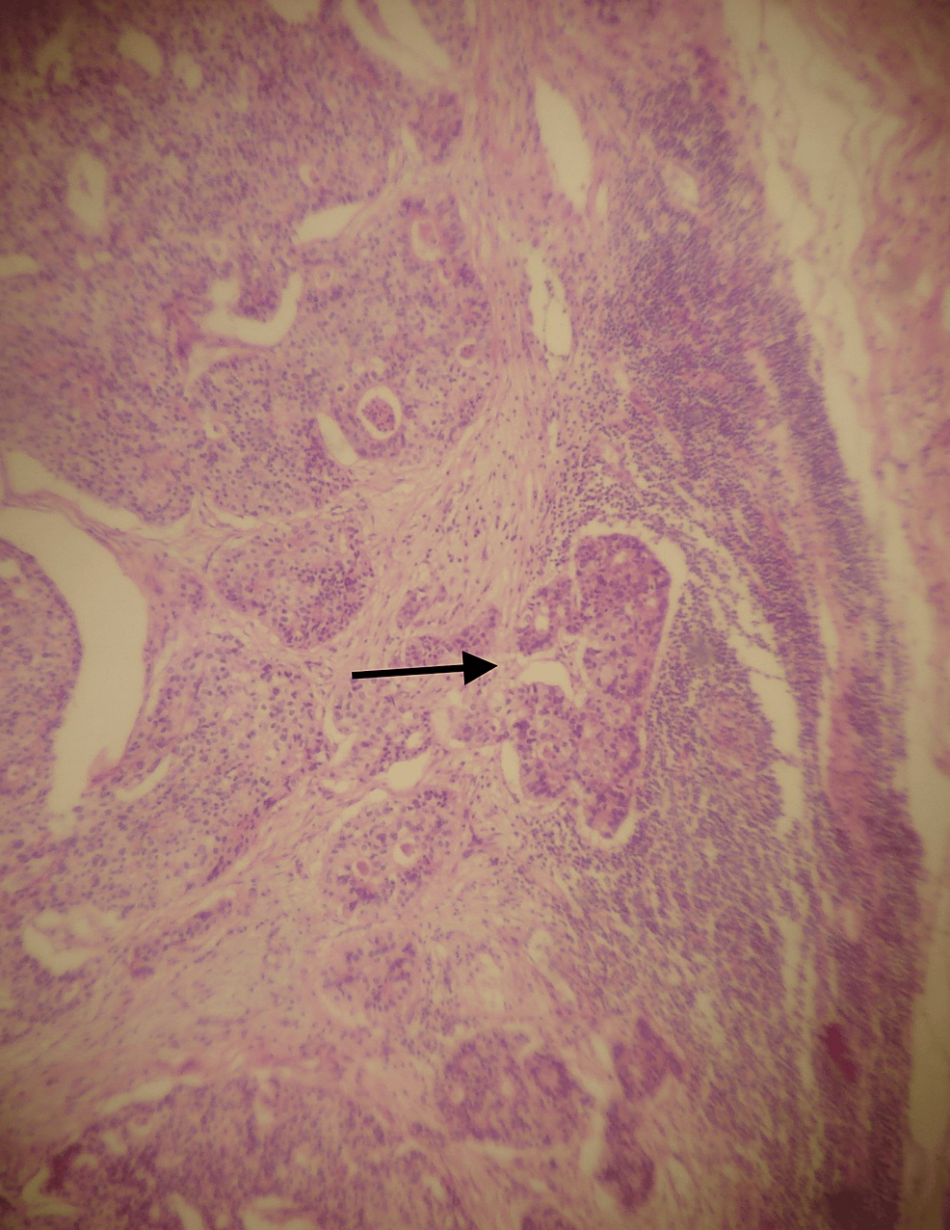
Histological image of the lymph node metastasis (arrow) (H & E x20)

The histopathology revealed a large bowel adenocarcinoma, measuring 3 x 2.5 x 2 cm, that had infiltrated the pericolic fat (Figure 3). In addition, six lymph nodes were found to be infiltrated (Figure 4).

The disease was staged as VIA (T4aN2aM1). The testis was not affected. The postoperative period was uneventful and the patient recovered fully. He was then referred to the oncology team but was subsequently lost to follow-up.

## Discussion

The existence of colon cancer within the hernia sac is extremely rare. Only a small number of such cases have been published worldwide, with approximately 23 cases published in the last 20 years (2003-2023) [7-9]. In a study by Zhang et al., the majority of the lesions were located within a left inguinal hernia (75%) and the most common pathology was sigmoid cancer (82.5%) [7]. The lesion was mainly located in the sigmoid colon [7]. Three additional cases where the lesion was identified in the cecum have been reported [8]. Our patient had a strangulated right inguinoscrotal hernia that had been present for several years and contained the caecum, ascending colon, and terminal ileum. Of note, 50% of the cases are only diagnosed intraoperatively [7]. Proper diagnosis is crucial to achieving optimal outcomes, and delayed or incorrect diagnosis carries the risk of incomplete tumor excision [6]. In our case, the diagnosis was suspected in the emergency setting preoperatively based on the CT of the abdomen and the pelvis.

The treatment should combine both hernia repair and careful oncological resection of the affected bowel, with or without anastomosis and stoma formation [6,7]. The operation can be performed with minimally invasive techniques; however, in the emergency setting of a strangulated hernia, open surgery is preferred [7]. The approach can involve either an abdominal or inguinal incision, or both [7]. The use of mesh is encouraged in the repair of scrotal hernias, except for cases with contamination, where suture repair may be preferred [10]. Shouldice repair with a non-absorbable monofilament suture could alternatively be performed [10]. Our patient underwent a right hemicolectomy, followed by tension-free herniorrhaphy. A mesh tension-free repair was discouraged due to the extensively contaminated field. Regarding postoperative mortality, emergency groin hernia repair is associated with a 30-day mortality that is 26 times higher compared to elective repair, whereas bowel resection in the emergency setting has a 30-day mortality rate of 7.9% [11].

Even though this condition is extremely rare, surgeons need to be mindful that longstanding inguinoscrotal hernias may coexist with cancer in the part of the colon within the hernia sac [9]. Clinicians should be alerted when a firm mass is palpated within the hernia during the examination [9]. In addition, anemia and weight loss should alert the surgeon that a tumor might also be involved in this presentation [9]. In such cases, a thorough investigation must be performed before surgery, whenever feasible [7].

## Conclusions

Proper and timely preoperative diagnosis is important to optimize the definitive management of this patient population and relies on clinical examination, detailed history taking, and appropriate imaging. The treatment protocol entails urgent intervention within the emergency setting, with the resection of the affected bowel. Subsequently, the procedure involves herniorrhaphy, either with or without the incorporation of a prosthetic mesh, as per the clinical state of the surgical field. This comprehensive approach aims to address both the primary malignancy and any concurrent hernia pathology, thereby optimizing patient management and therapeutic outcomes.

## References

[1] Tran H (2018). Endorsement of the HerniaSurge guidelines by the Australasian Hernia Society. Hernia.

[2] Patel VH, Wright AS (2021). Controversies in inguinal hernia. Surg Clin North Am.

[3] Perez AJ, Campbell S (2022). Inguinal hernia repair in older persons. J Am Med Dir Assoc.

[4] Rawla P, Sunkara T, Barsouk A (2019). Epidemiology of colorectal cancer: incidence, mortality, survival, and risk factors. Prz Gastroenterol.

[5] Jia SN, Han YB, Yang R, Yang ZC (2022). Chemokines in colon cancer progression. Semin Cancer Biol.

[6] Sabra H, Alimoradi M, El-Helou E, Azaki R, Khairallah M, Kfoury T (2020). Perforated sigmoid colon cancer presenting as an incarcerated inguinal hernia: a case report. Int J Surg Case Rep.

[7] Zhang J, Tang Y, Wu X, Wang G, Li T (2022). Sigmoid colon cancer masquerading as a right incarcerated inguinal hernia: a case study and literature review. Front Surg.

[8] Chern TY, Tay YK, Perera DS (2018). A rare case of ascending colon adenocarcinoma incarcerated in an inguinoscrotal hernia: case report and literature review. Surg Case Rep.

[9] Grossi U, Santoro GA, Tagliente G, Zanus G (2021). Sigmoid colon adenocarcinoma incarcerated in an inguinoscrotal hernia: diagnostic and management challenges. ANZ J Surg.

[10] Tran HM, MacQueen I, Chen D, Simons M (2023). Systematic review and guidelines for management of scrotal inguinal hernias. J Abdom Wall Surg.

[11] Sæter AH, Fonnes S, Rosenberg J, Andresen K (2022). Mortality after emergency versus elective groin hernia repair: a systematic review and meta-analysis. Surg Endosc.

